# Immunological mechanisms and therapeutic targets of fatty liver diseases

**DOI:** 10.1038/s41423-020-00579-3

**Published:** 2020-12-02

**Authors:** Hua Wang, Wajahat Mehal, Laura E. Nagy, Yaron Rotman

**Affiliations:** 1 Department of Oncology, the First Affiliated Hospital of Anhui Medical University, Hefei, Anhui China; 2 Inflammation and Immune Mediated Diseases Laboratory of Anhui Province, Hefei, Anhui China; 3 West Haven VA Medical Center, West Haven CT and Yale University School of Medicine, New Haven, CT USA; 4 Department of Inflammation and Immunity, Cleveland Clinic, Cleveland, OH USA; 5 Department of Gastroenterology and Hepatology, Center for Liver Disease Research, Cleveland Clinic, Cleveland, OH USA; 6 Department of Molecular Medicine, Case Western Reserve University, Cleveland, OH USA; 7 Liver and Energy Metabolism Section, Liver Diseases Branch, National Institute of Diabetes and Digestive and Kidney Diseases, National Institutes of Health, Bethesda, MD USA

**Keywords:** ALD, NAFLD, inflammation, cytokine, target, immune, Translational immunology, Immunotherapy

## Abstract

Alcoholic liver disease (ALD) and nonalcoholic fatty liver disease (NAFLD) are the two major types of chronic liver disease worldwide. Inflammatory processes play key roles in the pathogeneses of fatty liver diseases, and continuous inflammation promotes the progression of alcoholic steatohepatitis (ASH) and nonalcoholic steatohepatitis (NASH). Although both ALD and NAFLD are closely related to inflammation, their respective developmental mechanisms differ to some extent. Here, we review the roles of multiple immunological mechanisms and therapeutic targets related to the inflammation associated with fatty liver diseases and the differences in the progression of ASH and NASH. Multiple cell types in the liver, including macrophages, neutrophils, other immune cell types and hepatocytes, are involved in fatty liver disease inflammation. In addition, microRNAs (miRNAs), extracellular vesicles (EVs), and complement also contribute to the inflammatory process, as does intertissue crosstalk between the liver and the intestine, adipose tissue, and the nervous system. We point out that inflammation also plays important roles in promoting liver repair and controlling bacterial infections. Understanding the complex regulatory process of disrupted homeostasis during the development of fatty liver diseases may lead to the development of improved targeted therapeutic intervention strategies.

## Introduction

**Fig. 1:**
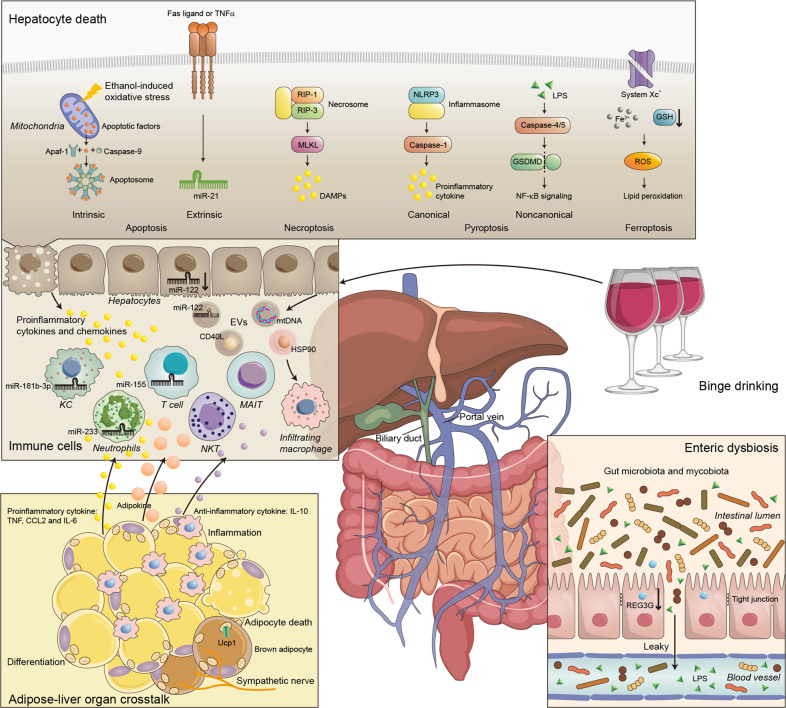
Triggers of the immune response in ALD. Dysregulated intestinal barrier integrity and gut microbiota products/metabolites play important roles in modulating ALD. The gut communicates with the liver via the gut–liver axis through the biliary system and the portal vein, transferring gut-derived components or the gut microbiota themselves to the liver and initiating the immune response. Chronic alcohol consumption disrupts the gut barrier, leading to increased gut permeability and ectopic immune stimulation. Chronic alcohol ingestion decreases intestinal REG3G expression, which is negatively associated with the number of mucosa-associated bacteria in both human patients and experimental mouse models. Alcohol exposure causes loss of epithelial cells at the tips of intestinal villi and a reduction in the levels of tight junction proteins. Adipose-liver organ crosstalk is mediated by the release of mediators, including neurotransmitters, cytokines, chemokines, adipocytokines, miRNAs, EVs, and metabolites, and the crosstalk between the liver and adipose tissue participates in promoting liver inflammation and injury in ALD. These mediators further activate immune cells, which release proinflammatory cytokines and chemokines, causing hepatocyte death

Excessive alcohol intake and high-calorie food consumption are two major etiological factors in the pathogeneses of chronic liver diseases worldwide, causing alcoholic liver disease (ALD) and nonalcoholic fatty liver disease (NAFLD), respectively. Although there are some differences in the hepatotoxicity caused by alcohol versus that due to excess caloric intake, immunological mechanisms play key roles in the pathogeneses of both ALD and NAFLD. With regard to ALD, although alcohol-induced hepatotoxicity and oxidative stress are the key components contributing to its pathogenesis, recent studies have clearly shown that the immune response might also substantially contribute to the development of ALD, including its inflammatory component, alcoholic steatohepatitis (ASH) (Fig. 1). The early working model for ALD initiation demonstrated that portal circulation of the bacterial product lipopolysaccharide (LPS) from alcohol-induced gut leakage to liver-activated Kupffer cells (KCs) through LPS/Toll-like receptor (TLR) 4 signaling and subsequently produced inflammatory cytokines such as tumor necrosis factor alpha (TNF-α), leading to alcoholic liver injury.^1–3^ In recent years, emerging evidence from preclinical and clinical studies has suggested that new immunological mechanisms are involved in all stages of ALD, including immune response initiation, inflammatory reactions, and completed/unresolved repair.^4^ In the early stage, inducers/sensors, including alcoholic hepatocyte death, cause an immune response in the liver. In the second stage, immune mediators interacting with immune cells lead to inflammation and hepatocyte death. Ultimately, the pathological consequences of the immune response associated with ALD include elimination of cell death debris and proliferation of hepatocytes, which may result in complete recovery or unsolved repair manifesting as fibrosis/cirrhosis. Intestinal microbes influence the immune response in the liver through pathogen-associated molecular patterns (PAMPs), and PAMPs further mediate the activation of innate immune cells through pattern recognition receptors.^5–7^ Moreover, the damaged liver produces damage-associated molecular patterns (DAMPs) and stimulates inflammatory signals.^6,8,9^ In addition, mechanisms of crosstalk between organs, including adipocyte death, promote the progression of ALD through the transmission of DAMPs or extracellular vesicles (EVs) with the migration of immune cells. In the first part of this review, we summarize the current understanding of the immunological mechanisms in ALD by discussing immune response triggers (such as enteric dysbiosis, hepatocyte death and adipose-liver organ crosstalk) and immune response courses, including multiple immune cell types, major immune pathways, and specific immune mediators. We also highlight possible therapeutic interventions for these immune responses in the treatment of ALD.

**Fig. 2:**
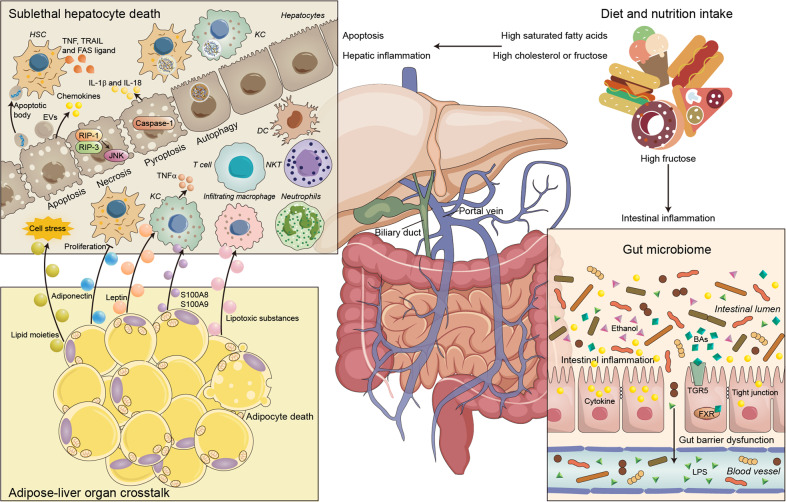
Triggers of the immune response in NAFLD. The gut microbiota plays a key role in the pathogenesis of NAFLD. Gut barrier dysfunction increases bacterial translocation and promotes NAFLD progression. The destruction of the intestinal vascular barrier by the microbiota causes bacteria or bacterial products to enter the blood circulation, which is a prerequisite for liver inflammation and the development of NASH. Crosstalk between adipose tissue and the liver affects systemic metabolism and insulin resistance. Adipose tissue plays a key role in regulating NASH development by secreting adiponectin, leptin, TNF, and IL-6. In addition, some lipid moieties (palmitic acid, ceramide) released by adipocytes also hinder the function of the ER and mitochondria, causing cell stress and even hepatocyte death. Hepatocyte death is one of the key triggers of liver inflammation in NAFLD and NASH progression. In addition, KCs produce TNF, TRAIL, and FAS ligands through phagocytosis of apoptotic bodies, which subsequently promotes hepatocyte apoptosis and causes hepatitis and fibrosis. The release of IL-1β and IL-18 into the circulation activates the immune system, and the alteration of autophagy in hepatocytes and nonparenchymal cells (KCs and HSCs) contributes to NASH pathogenesis

NAFLD is becoming a major cause of liver-related morbidity worldwide, impacting nearly 25% of the global population,^10,11^ and is typically associated with obesity, insulin resistance, diabetes, and dyslipidemia.^12^ NAFLD can manifest as nonalcoholic fatty liver (NAFL), also termed simple steatosis, or as nonalcoholic steatohepatitis (NASH), a more severe form where fat accumulation is accompanied by inflammation and injury and is related to an increased risk of the development of cirrhosis or hepatocellular carcinoma (HCC).^13,14^ Recent findings support the “multiple-hit” hypothesis of the pathogenesis of NAFLD, which states that systemic changes such as liver, intestinal tract, and adipose tissue changes lead to the development of NAFLD.^15,16^ Recently, these concurrent exogenous and endogenous hits have been investigated as potential therapeutic targets.^17^ Inflammation is one of the main pathogenic factors of NASH.^15^ In the second part of this review, we will probe the latest concepts regarding the roles of various inflammatory cells in the occurrence and development of NAFLD and discuss potential immune cell-targeted therapies for NAFLD (Fig. 2).

## Immunological mechanisms of ALD

### Triggers of the immune response in ALD

#### Enteric dysbiosis

The mammalian intestine contains a variety of microbes (bacteria, archaea, fungi, and viruses) and expresses over 3 million genes.^18^ The gut microbiota is crucial for the maintenance of intestinal barrier integrity, regulation of gut homeostasis, and stimulation of the host immune response.^19^ The direct roles of enteric dysbiosis in the initiation and development of ALD have become much clearer in the past decade. Dysregulated intestinal barrier integrity and gut microbiota products/metabolites play important roles in modulating ALD. The gut communicates with the liver via the gut–liver axis through the biliary system and the portal vein, transferring gut-derived components or the gut microbes themselves to the liver and initiating the immune response.^6^ Mice treated with a broad-spectrum antibiotic cocktail (Abx) are resistant to alcohol-induced liver injury and neuroinflammation.^20^ In addition, administration of probiotics such as *Lactobacillus rhamnosus GG* has been found to alleviate ALD in a mouse model.^21^ Interestingly, ASH can be transmitted from patients to healthy germ-free and conventional mice via intestinal microbiota transplantation.^22^ Fecal microbiota manipulation with transplantation of fresh feces from alcohol-resistant donor mice into alcohol-sensitive recipient mice prevents steatosis and liver inflammation and restores aspects of gut homeostasis, such as the *Bacteroides* proportion.^23^ In patients with alcoholic hepatitis (AH), increased abundance of cytolytic *Enterococcus faecalis* is closely associated with increased severity of clinical outcomes and increased mortality. Cytolysin secreted by *E. faecalis* is responsible for hepatocyte death and liver injury in both AH patients and mouse models. Bacteriophages that target cytolytic *E. faecalis* decrease cytolysin levels in the liver and abolish ethanol-induced liver disease in humanized mice.^24^ Intestinal fungi have also recently been reported to be involved in human ALD. Patients with ALD have lower fungal diversity than controls and exhibit overgrowth of Candida. In addition, a stronger systemic immune response to fungal products or fungi is associated with increased mortality in patients with AH.^25^ Chronic alcohol consumption induces intestinal *mycobiota* overgrowth and translocation of fungal β-glucan into the systemic circulation in mice. Treatment with the antifungal agent amphotericin B successfully attenuates features of ethanol-induced liver disease in mice.^26^

Chronic alcohol consumption disrupts the gut barrier, leading to increased gut permeability, and ectopic immune stimulation. Under conditions of gut homeostasis, multiple barriers protect the human body from invading microbes; for example, commensal bacteria inhibit the colonization of pathogens.^6^ To protect the first physical barrier separating the gut lumen and the host, both intestinal epithelial cells and Paneth cells secrete antimicrobial proteins to suppress the translocation of bacteria to the inner mucus layer. These cells can also secrete a c-type lectin, regenerating islet-derived 3 gamma (REG3G), to maintain the spatial segregation of the microbiota and host. Chronic alcohol ingestion decreases intestinal REG3G expression, which is negatively associated with the number of mucosa-associated bacteria in both human patients and experimental mouse models.^27^ Alcohol exposure causes loss of epithelial cells at the tips of intestinal villi and reductions in the levels of tight junction proteins (e.g., occludin and ZO-1). Alcohol-induced local inflammation, oxidative stress, circadian rhythm disruption, and malnutrition further contribute to intestinal tight junction damage.^28^ Disruption of barrier integrity by alcohol seems to be a persistent event because elevated levels of permeability markers such as ^51^Cr-ethylenediaminetetraacetic acid (^51^Cr-EDTA) have been detected in alcoholic cirrhosis patients even after 2 weeks of abstinence.^29^ Translocation of bacteria or microbial products through the gut mucosa to the liver is a critical pathological event for the transition from alcoholic steatosis to ASH. Intestinal antimicrobial molecules are dysregulated following chronic alcohol feeding, contributing to enteric microbiome changes and to ASH.^30^ The early hypotheses regarding ALD were focused on elevations in blood LPS levels in both ALD patients and animal ALD models and more importantly, on the strong correlations between LPS levels and the severity of ALD (e.g., the levels are much higher in the alcoholic cirrhosis stage than in other stages of ALD).^31^ Toll-like receptor 4 (TLR4) on KCs is one of the direct interacting targets of LPS. Upon activation, inflammatory cytokines and chemokines attract neutrophils and monocytes to the liver. Deficiencies in TLR4 or LPS-binding proteins ameliorate alcohol-induced liver injury in mouse models.^32,33^

#### Hepatocyte death in ALD

In response to alcohol intoxication, distinct types of hepatocyte death, including apoptosis, necroptosis, pyroptosis, and ferroptosis, determine the severity of inflammation as well as the different spectra of ALD. The mitochondrial apoptotic pathway activated by ethanol- or acetaldehyde-induced oxidative stress involves the release of several apoptotic factors (e.g., cytochrome c and apoptosis-inducing factor) into the cytosol. These factors bind to Apaf-1 and caspase-9 to form the “apoptosome” to trigger the intrinsic apoptotic pathway, which is generally considered to be noninflammatory.^34^ It has been demonstrated that ethanol exposure increases Fas ligand- and TNF-α-mediated extrinsic hepatocyte apoptosis partly via miR-21. This process seems to contribute to the pathogenesis of AH.^35,36^ Recent studies have highlighted the vital roles of necroptosis, characterized by morphological changes such as cell volume augmentation, plasma membrane disruption, and cellular collapse, in ALD progression facilitation. Necroptotic cells release a variety of DAMPs to trigger an inflammatory response in the liver.^37^ Receptor-interacting protein (RIP)-1 and RIP-3 are central triggers of necroptosis that induce the process partly via the formation of a necrosome complex and subsequent mixed-lineage kinase domain-like (MLKL) signaling.^38^ Knockout of RIP-3 protects mice from alcohol-induced hepatic steatosis and inflammation.^39,40^ A recent study also reported that MLKL-dependent but RIP3-independent signaling contributes to NASH-induced liver injury via autophagic inhibition.^41^ Whether similar signaling can be phenocopied in ALD models warrants further investigation. Both PAMPs and DAMPs produced in or transported to the liver after ethanol consumption can activate hepatocyte pyroptosis, another important kind of programmed cell death. Both canonical and noncanonical pyroptosis contribute to ALD pathogenesis. Canonical pyroptosis relies on caspase-1 and is mediated by inflammasomes (e.g., the NLRP3 inflammasome). In chronic-binge model mice, alcohol inhibits mitophagy to induce NLRP3 inflammasome assembly and proinflammatory cytokine production in hepatocytes. Targeting this pathway effectively attenuates alcoholic liver injury.^42^ Noncanonical pyroptosis signaling can be directly activated by LPS independent of TLR4. Mechanistically, activated caspase-11 (mice) or caspase-4/5 (humans) senses intracellular LPS to cleave gasdermin D (GSDMD) within its linking loop. Then, it binds to phosphoinositides on the plasma membrane and lyses the membrane to cause cell death.^43^ GSDMD plays a key role in the pathogenesis of NASH by regulating lipogenesis, the inflammatory response, and the NF-ĸB signaling pathway. Caspase-4/11 and GSDMD are commonly upregulated in AH mice and patients. Caspase-11 deficiency reduces GSDMD activation, liver inflammation and bacterial load, while hepatocyte-specific activation of GSDMD aggravates hepatocellular lytic death and leukocyte inflammation.^44^ Iron overload is commonly observed in ALD patients. Chronic alcohol consumption causes elevations in serum ferritin concentrations and transferrin saturation, resulting in increases in hepatic iron stores. As an iron-dependent oxidative programmed cell death mechanism, ferroptosis generates oxygen species and local inflammation in the liver.^45^ It also features glutathione (GSH) depletion, glutamate antiporter (system Xc-) disruption, and lipid hydroperoxide overexpression.^46^ Alcohol administration induces excessive iron accumulation and ferroptosis in both patients and animal models. Several novel ferroptosis pathways have been identified in ALD models. For example, intestine-specific sirtuin 1 (SIRT1) deletion substantially attenuates hepatic inflammation and liver injury by reducing iron accumulation, increasing GSH levels, and inhibiting a panel of genes implicated in the ferroptosis process in the liver.^47^ In adipose tissue, overexpression of lipin-1, which is a Mg^2+^-dependent phosphatidic acid phosphohydrolase involved in the generation of diacylglycerol during the synthesis of phospholipids and triglycerides, contributes to ethanol-induced hepatic ferroptosis.^48^

Finally, damaged or stressed hepatocytes can release a large number of proinflammatory mediators, including cytokines and chemokines, thereby promoting liver inflammation in ALD. Knockout of IL-17RA downregulates the expression of CXCL1 and other chemokines, which delays the development of ALD in liver cancer.^49^ Analogously, serum and hepatic levels of CCL2, MCP-1 and macrophage migration inhibitory factor (MIF) are increased in ALD patients, and these chemokines produced by hepatocytes play significant roles in immune cell recruitment and inflammatory activation in ALD.^49–51^ MIF is released from hepatocytes during ethanol-induced liver injury rather than from immune cells such as macrophages.^52^ MIF is an important danger signal released by liver cells under ethanol stimulation, and it has a protective effect against hepatocyte steatosis.^51,53^ The chemokine receptors CXCR2 and CXCR4 are functional receptors of MIF and can form receptor complexes with CD74. MIF activates downstream signaling cascades by binding to the receptor complex and plays vital roles in regulating liver immune cell recruitment and liver injury.^51^ In addition, MIF is expressed in the liver earlier than MCP1 and can regulate the expression of MCP-1 in injured liver tissue through autocrine CD74, CD44, and p38 MAPK signaling.^54^

#### Binge drinking

Since Dr. Gao’s group at NIH developed a chronic-plus-binge ethanol feeding model (the NIAAA model, later also called the Gao-Binge model) in 2013,^55^ researchers have demonstrated that ethanol bingeing induces significant hepatic neutrophil infiltration and liver injury in mice chronically fed ethanol via either ad libitum feeding^56^ or continuous intragastric feeding.^57^ In addition, circulating neutrophil levels are markedly higher in individuals with alcoholism who have recently engaged in excessive binge drinking than in those who have not recently engaged in binge drinking.^57^ Moreover, the number of circulating neutrophils correlates well with the serum ALT level in individuals with alcoholism, suggesting that elevated neutrophil levels contribute to liver damage in these individuals.^57,58^ Mechanistically, binge ethanol intake causes hepatocyte damage, which induces the release of mitochondrial DNA (mtDNA)-enriched EVs. These EVs subsequently activate neutrophils and induce hepatic neutrophil infiltration.^59^ Neutrophils can produce reactive oxygen species (ROS), subsequently activating stress kinases (e.g., ASK1 and p38 MAPK), and induce alcoholic liver injury.^60^

#### Adipose-liver organ crosstalk

The normal function of adipose tissue is profoundly influenced by excessive alcohol ingestion, causing local inflammation and changes similar to those seen in obese patients. Different drinking patterns or different types of alcoholic beverages may have different effects on body mass and adiposity. For instance, beer and spirit drinking usually leads to greater weight gain than wine drinking.^61^ Alcohol also directly provokes adipocyte death and adipose tissue inflammation.^62^ Differentiation of preadipocytes and production of adipokines by adipocytes are also disrupted after ethanol consumption.^63–65^ These processes cause resistance of adipose tissue to insulin, increase lipolysis, and lead to production of proinflammatory cytokines in the liver.^66^ In particular, alcohol promotes TNF, CCL2, and IL-6 production in adipose tissue, and the levels of these molecules are correlated with the severity of ASH in patients.^67^ Release of the anti-inflammatory cytokine IL-10 from adipose tissue is also provoked by alcohol as a strategy to compensate for liver injury.^68^ In addition, various types of inflammatory cells in adipose tissues, such as macrophages, dendritic cells (DCs), neutrophils, and T/B cells, are modulated by alcohol ingestion and the presence of TLR4.^69^ Moreover, alcohol intake alters the adipokine secretion of leptin, visfatin, resistin, and adiponectin to activate both KCs and hepatic stellate cells (HSCs), resulting in accelerated liver inflammation and fibrosis.^65,70^ A recent study also found that alcohol consumption or direct alcohol administration into the brain increases brown adipose tissue uncoupling protein 1 (Ucp1) expression and activity in a brown adipose tissue sympathetic nerve-dependent manner. Genetic deletion of Ucp1 exacerbates alcohol-induced liver steatosis, injury, inflammation, and fibrosis in mice.^71^ It should be noted that alcohol and obesity have synergistic effects on liver injury, since a clinical study has revealed that obese men who drink 15 or more units of alcohol a week have a substantially higher risk of liver-related mortality than subjects with a single disease.^72,73^ Moreover, adipocyte death itself predominantly induces liver injury and inflammation in a chemokine (C–C motif) receptor 2-positive (CCR2^+^) macrophage-dependent manner and enhances the sensitivity of hepatocytes to lipotoxicity.^74^ Undoubtedly, crosstalk between the liver and adipose tissue participates in promoting liver inflammation and injury in ALD; however, the underlying mechanisms need to be explored for future clinical considerations.^75^

### Immune cells in the pathogenesis of ALD

#### Macrophages

Liver macrophages consist of tissue‐resident macrophages (KCs) and infiltrating macrophages. KCs exist within the hepatic sinusoids in healthy liver tissue, representing the largest population of liver-resident immune cells that scavenge microbial products in the blood from the intestine. Alcohol-induced sensitization of liver macrophages to portal LPS is considered a key mechanism of steatosis, injury/inflammation, and fibrogenesis in ALD.^76^ In general, both resident (KCs) and infiltrating macrophages exhibit strong plasticity, regulating signals within their immune microenvironment in the liver.^77^ KCs play a pivotal role in the inflammatory response accompanying the progression of ALD.^78^ Hepatic macrophage populations are increased in ALD, and macrophages derived from infiltrating monocytes are thought to contribute to this expansion. Activated inflammasomes and IL-1β drive the pathogenesis of ALD, and this pathogenic effect is KC specific.^79^ Furthermore, IL-17A critically regulates alcohol-induced hepatic steatosis, inflammation, fibrosis, and HCC. Genetic deletion or pharmacological blockade of Th17 cells visibly reduces liver injury and further development of HCC by inhibiting KC activation and decreasing cholesterol synthesis in fatty liver cells.^49^ In early ALD, ST2 inhibits hepatic macrophage activation through NF-κB to protect against injury, and IL-33 is not involved in the immune response to ALD. However, in the late stage of ALD aggravation, massive cell death accompanied by IL-33 release triggers IL-33/ST2 signaling and subsequently promotes tissue damage.^8^ TLRs also play key roles in the progression of ALD. TLR4 expressed on immune cells and parenchymal cells recognizes LPS, activates downstream signaling cascades and induces the activation of proinflammatory cytokines.^32^ In addition, a study has shown that upon chronic alcohol intake, mitochondrial double-stranded RNA (mtdsRNA) is produced and enriched in exosomes in the liver, and TLR3 activation by mtdsRNA released from exosomes triggers the production of IL-1 by neighboring KCs, thereby inducing liver inflammation in ALD.^80^

According to their differences in Ly6C expression, infiltrating macrophages can be further divided into two subgroups. Ly6C^low^ cells show an anti-inflammatory and tissue-protective phenotype; in contrast, Ly6C^hi^ cells show a proinflammatory, tissue-damaging phenotype.^81^ Ly6C^hi^ infiltrating macrophage populations are increased in ALD, significantly enhancing liver injury. Ly6C^hi^-infiltrating macrophages can become Ly6C^low^-infiltrating macrophages after apoptotic hepatocytes are removed. Treatment with the dual CCR2/5 inhibitor cenicriviroc (CVC) can reduce Ly6C^hi^ macrophage numbers in addition to reducing the total number of macrophages infiltrating the liver. Proinflammatory cytokine expression and macrophage infiltration are inhibited after CVC treatment, suggesting the involvement of a CCR2-dependent mechanism of macrophage migration in ALD.^82^ Thus, the two different types of infiltrating macrophages that are recruited to the liver likely play different or even opposite roles in regulating alcoholic liver inflammation and repair.^83^ In a recent publication, a fraction of KCs derived from Ly-6C^+^ monocytes was reported during NASH with underlying impairment of self-renewal ability. These monocyte-derived KCs exacerbated liver damage, highlighting functional differences among KCs with different origins.^84^ Whether such a specific macrophage/monocyte subpopulation also contributes to ALD via a similar mechanism has yet to be studied.

#### Neutrophils

Neutrophils are the most abundant innate immune cells in the human body, accounting for ~40–75% of circulating human white blood cells. Granulocyte colony-stimulating factor (G-CSF) is the main regulator of neutrophil development, production and release in bone marrow. Furthermore, IL-23 produced by macrophages activates T-helper cells to release IL-17, which in turn increases the production/release of neutrophils via G-CSF.^85^ TLR2 and TLR9 participate in alcohol-mediated liver injury by inducing CXCL1 expression and neutrophil infiltration.^86^ Mature neutrophils reside in the bone marrow due to the CXCR4 signal triggered by CXCL12 on stromal cells, while CXCR2 promotes their release from the bone marrow into the circulation to perform physiological functions. The severity of ALD is related to the number of neutrophils in the liver. Neutrophils also play important roles in the regulation of intestinal flora and bacterial infection in ALD due to their killing and phagocytosis of pathogenic microorganisms.^87^

Circulating neutrophils are defective in the context of ALD, but this defect can be reversed by restoring the IL-33/ST2 pathway.^88^ In addition, removing neutrophils can alleviate LPS-induced systemic inflammation and liver damage in ALD.^89^ However, in a clinical study and death risk assessment of 121 patients, the 90-day mortality rate was independently related to the degree of fibrosis, neutrophil infiltration, bilirubin balance type, and presence of giant mitochondria in ALD patients, and neutrophils may secrete cytokines to stimulate liver regeneration.^90^ Similarly, G-CSF improves the function of normal and dysfunctional granulocytes, which also secrete cytokines to stimulate liver regeneration, and mobilizes hematopoietic stem cells to promote their differentiation and function, which can protect against liver injury and improve survival rates in ALD patients.^91^ However, further preclinical data are still needed to support the theory of neutrophil therapy. In addition, lipocalin-2 (LCN2) is an iron group-binding peptide in neutrophils that plays a role in tissue protection during the inflammatory response. Alcohol-fed mice show increased hepatic LCN2 expression that is mainly concentrated on neutrophils. Compared with wild-type controls, *Lcn2*^−/−^ mice have reduced neutrophil infiltration, liver injury, and liver fatty degeneration, indicating that *Lcn2* deletion has a protective effect against ALD.^92^ In human AH patients, ductular reaction (DR) has a proinflammatory profile and promotes neutrophil recruitment, which indicates that DR may be involved in the liver inflammatory response in AH and may provide a potential target for ALD treatment.

On the one hand, neutrophils directly cause inflammation development and hepatocyte damage in ALD. Moreover, the cytokines released by neutrophils are important mediators for the regulation of inflammation and tissue repair.^89–91,93^ Thus, in ALD, the functions of neutrophils are complex and multifaceted. In future studies, special attention should be paid to the complexity and diversity of neutrophil functions to develop targeted interventions and treatment strategies for ALD.

#### T cells

T cells are also involved in the pathogenesis of ALD, as key chemokines such as CCL5 are upregulated in these cells in the liver.^94^ During the differentiation and maturation of T cells, various membrane proteins (such as T-cell receptor (TCR), CD3, CD4, and CD8) are expressed on the cell surface. These proteins recognize antigens and mediate specific immune responses and immune regulation. In the livers of patients with ALD, the populations of both CD4^+^ and CD8^+^ T cells are increased.^95^ Similarly, the numbers of CD4^+^CD57^+^ and CD8^+^CD57^+^ T cells in the peripheral blood of patients with ALD are increased, and mice with chronic ethanol consumption have higher levels of CD44 expression in CD8^+^ T cells than control mice.^96^ Adducts produced during ethanol metabolism have been identified in the livers of ALD patients; these adducts can be presented to CD4^+^ T cells by antigen-presenting cells (APCs), thereby inducing clonal T-cell proliferation.^97^ A recent study on differential TCR characteristics identified by high-throughput sequencing has indicated that liver-infiltrating T cells in ALD exhibit reduced polyclonality. Simultaneous detection of ALD-related clonotypes may attest to the presence of neoantigen-specific T-cell responses in ALD that contribute to the pathogenesis of ALD.^98^

Different types of specific T cells play different roles in regulating ALD. T cells are related to liver inflammation, necrosis, and regeneration in ALD patients, indicating that T cells may not only promote disease progression by releasing inflammatory mediators (such as TNF-α, IL-1, and IL-17)^99–101^ and directly harm hepatocytes through cytotoxic CD8^+^ T lymphocytes^99,102^ but also play a beneficial role in ALD by reducing inflammation and promoting liver regeneration.^103,104^ For example, the intensity of the Th1 cell response is directly related to the severity of the disease. Alcohol dehydrogenase (ADH) peptide induces the production of IFN-γ, IL-4, and IL-17. IL-4 production in excessive drinkers is lower than that in active abstainers, while IL-17 and IFN-γ production is higher in excessive drinkers.^104^ Th17 cells are crucial in the pathogenesis of ALD. Reducing Th17 cell numbers in the gut can reduce liver damage,^105^ but Th17 cells may also secrete IL17 to promote liver damage repair.^106^ In addition, a recent study has indicated that IL-17A is produced mainly by γδ T cells after ethanol bingeing, while IL-17A is produced mainly by CD4^+^ T cells in mice after acute or chronic alcohol consumption.^107^ Interestingly, the ethanol metabolite acetaldehyde has been shown to inhibit T-cell glucose metabolism and functions, which may contribute to increased incidences of bacterial infection in individuals with alcoholism.^108^

#### Natural killer T (NKT) cells

NKT cells express surface receptors of NK cells (such as NK1.1 in mice and CD161^+^/CD56^+^ in humans) and TCR, which is unique to traditional T cells and recognizes lipid antigens through CD1d (an MHC class 1 molecule). NKT cells in the liver respond very quickly to injury, either directly by identifying related lipids or indirectly through secretion of TLR ligands and cytokines (such as IL-12, IL-4, and IFN-γ) by activated APCs such as KCs, hepatocytes, and myeloid DCs.^100,109,110^ Furthermore, type I NKT cell-induced inflammation and neutrophil recruitment lead to liver tissue damage, while type II NKT cells have a protective effect against ALD damage. Type I NKT cells are activated after alcohol intake, and inhibition of type I NKT cells by retinoic acid or sulfonamide can prevent ALD.^111^

#### Mucosa-associated invariant T (MAIT) cells

MAIT cells are widely distributed in the liver, blood, and intestinal mucosa and are key components of antibacterial defense. MAIT cells usually account for 1% of T cells in mouse tissues but are more abundant in human tissues, usually representing 45% of human liver lymphocytes and 2% of T cells in human blood. MAIT cells express the traditional TCR, which can recognize the microbial-derived vitamin B metabolites presented by MHC-related 1 (MR1).^112,113^ In ALD, decreased numbers and dysfunction of MAIT cells lead to a higher frequency of acquired bacterial infections.^114^ During the development of ALD, reprogramming MAIT cells with IL-15 can enhance their antibacterial activity and prevent tissue damage mediated by the activation of MAIT cells.^100^ Therefore, MAIT cells likely contribute to the pathogenesis of human ALD,^114^ but more studies are needed to confirm this possibility.

### Emerging mechanisms in ASH

#### MicroRNAs (miRNAs)

MiRNAs, which are a class of highly conserved single-stranded RNAs, bind to the 3′-untranslated regions (UTRs) of target RNAs through complementary base pairing and subsequently inhibit the expression of their target genes. In addition, miRNAs can inhibit the protein expression of targeted mRNAs through mRNA degradation. Recent studies have demonstrated that many miRNAs can silence the expression of inflammatory factors and affect immune response pathways to regulate the process of ASH.^115^ Serum miR-122, miR-223, miR-155, and miR146a levels are elevated in ALD.^116,117^ Hepatocyte-specific miRNA-122 protects the liver against inflammation by reducing hepatic expression of HIF1α, but miRNA-122 expression is downregulated in ALD.^118^ The levels of neutrophil-specific miR-233 in the serum and liver are increased in both ALD patients and animal models; this miRNA plays an important role in inhibiting neutrophil overactivation by targeting the IL-6-p47^Phox^ pathway in neutrophils.^58^ MiR-155 exerts proinflammatory effects in ASH, while miR-181b-3p exerts an anti-inflammatory effect via inhibition of KCs.^119,120^ In addition, alcohol-mediated dysregulation of the miR181b-3p-importin α5 regulatory axis in hepatic macrophages leads to the sensitization of KCs to TLR4 stimulation, resulting in liver inflammation in ALD.^120^

#### EVs

EVs are nanoscale membrane-derived vesicles and include exosomes, microvesicles, and apoptotic vesicles. The cargos in EVs include proteins, lipids, nucleic acids, and metabolites that vary with changes in the physiological environment, thus regulating the transcription or metabolism of target cells. Alcohol can promote exosome secretion and inhibit autophagy flux. MiR-155 plays a key role as a mediator in the crosstalk between autophagy and the secretion of exosomes. Hepatocytes treated with alcohol release exosomes containing miR-122, which inhibits the heme oxygenase-1 (HO-1) pathway and subsequently increases LPS sensitivity, resulting in hepatocyte injury and causing ASH.^121^ In addition to miRNAs, EVs can also transfer RNA, DNA, protein and other molecules into target cells. With the assistance of caspase-3, EVs containing CD40L bind with homologous receptors expressed on macrophages, thus promoting M1 macrophage polarization in ALD.^122^ Similarly, HSP90 in EVs also contributes to the activation of macrophages in ALD.^123^ Mitochondrial RNA (mtRNA)-enriched EVs contribute to the recruitment of neutrophils via TLR9,^59^ while activation of ASK1 and p38MAPKα plays an important role in controlling the release of mtDNA-enriched EVs in ALD.^60^ EVs containing mtDNA also stimulate the production of IL-17 via TLR3 after alcohol treatment.^80^

#### Complement

Complement is an intrinsic component of the innate immune system that is linked to the activation of adaptive immunity. Complement is activated by 3 pathways, the classical, lectin, and alternative pathways, resulting in the generation of the anaphylatoxins C3a and C5a.^124^ The role of complement in ALD is complex; some components of the complement pathway contribute to injury, while others are protective. For example, in murine models of ALD, both C3 and C5 contribute to injury, while the complement regulator CD55 protects against injury.^125,126^ Similarly, C1q, a component of the classical pathway, contributes to injury,^127^ while Factor D, essential for the alternative pathway, offers protection, likely via removal of cellular debris.^128^ Adding to the complexity, complement receptors, including C5aR1, can have cell-specific roles in murine models of ALD.^129^ There is evidence of complement activation in both the liver and circulation of patients with ALD,^130,131^ and reduced concentrations of complement factor I and soluble C5b9 are associated with an increased risk of mortality in patients with severe AH.^131^

### Inflammatory therapeutic targets for the treatment of ALD

Because of the clear contribution of inflammation to the progression of ALD, a number of therapeutic targets are being investigated with the goal of interrupting the nonresolving inflammation associated with ALD. AH in its most severe form has a 30-day mortality rate on the order of 40%.^132,133^ The current approved therapy involving prednisolone is effective in only a minority of patients. While early studies on the success of early liver transplantation are promising,^134^ surgical interventions are extremely invasive and expensive. Therefore, the development of effective therapeutics for AH is an important unmet clinical need. Despite this need, there have been relatively few clinical trials addressing AH; clinicaltrials.gov (September 2020) lists 88 registered clinical trials. Only 37 of these have been completed, and as few as 7 trials have posted results.

Preclinical experiments in murine models of ALD have identified a number of potential therapeutic targets. Here, we will review some of these targets, including those aimed at normalizing gut dysbiosis and improving the intestinal barrier, reducing oxidative stress and hepatocyte death, and interrupting the production and/or signaling capacity of inflammatory cytokines and chemokines.

### Microbial dysbiosis and intestinal barrier function

The microbial dysbiosis that accompanies ALD has been well described, but efforts to normalize dysbiosis are just beginning.^135,136^ Early studies identified the roles of gut microbes in ALD using nonabsorbable antibiotics. In murine models of ALD, treatment with probiotics, such as LGG, and synbiotics has shown efficacy in preventing ethanol-induced liver injury.^21,137–139^ Similarly, fecal transplant studies in mice have shown some promise.^135^ To date, there are limited data on the efficacy of nonabsorbable antibiotics, probiotics or fecal transplants in patient populations; however, one study did find that fecal transplants improved outcomes in patients with alcohol use disorder (AUD).^140^

A variety of nutritional supplements have also been tested in murine models for their ability to improve intestinal integrity and limit the transfer of PAMPs to the portal circulation and liver. Related strategies have included treatment with butyrate, an important fuel source for colonic enterocytes, as well as multiple molecules shown to improve tight junction integrity in the intestine, such as zinc, saturated fatty acids, glutamine, and hyaluronic acid with an average molecular weight of 35 kDa (HA35).^141–145^ To date, only zinc supplementation has been tested in patients with AH, used in combination with anakinra and pentoxifylline as part of a large multicenter clinical trial.^146^

### Hepatocyte injury

While PAMPs entering the portal circulation from the gut are one source of inflammatory signals contributing to ALD, DAMPs derived from injured or dead cells are other potential targets for therapeutics in ALD. In this regard, investigators have taken the approach of either improving the health of hepatocytes via treatment with oxidative stress-reducing agents or decreasing hepatocyte death.^147,148^ For example, supplementation of mice with N-acetylcysteine reduces ethanol-induced oxidative stress in hepatocytes,^149^ but small clinical trials have not found long-term beneficial effects.^147^ Current studies are testing whether mitochondrial-targeted antioxidants might be more therapeutically useful than general antioxidants.^147^

Hepatocyte death is another potential therapeutic target. However, hepatocytes can undergo cell death via multiple pathways, including apoptosis, necroptosis, pyroptosis, and ferroptosis.^36,150^ Early studies in mice suggested that inhibition of apoptosis, either pharmacological or genetic, does not prevent ethanol-induced inflammation and hepatocyte injury but does reduce the development of fibrosis.^151^ More recent studies implicating RIP3 and GSDMD in ethanol-induced hepatocyte cell death have helped explain why apoptosis prevention alone is not protective.^40,44,152^ However, there are few available therapeutic agents that target the other modes of cell death. Complementary strategies to promote hepatocyte regeneration are also being explored. For example, several groups are interested in the potential therapeutic properties of G-CSF, a potent growth factor proposed to promote hepatocyte regeneration.^136,153,154^ IL-22 is a hepatoprotective cytokine that has been shown to protect against alcoholic hepatitis through multiple targets.^106,155^ Recently, an open-label, cohort dose-escalation phase IIa study revealed that treatment of patients with moderate and severe alcoholic hepatitis with IL-22 was safe and showed an improved mortality rate and clinical manifestations.^156^

### Therapeutics to directly reduce inflammation

By far, the most studied therapeutic avenue for ALD and in particular for AH is the use of anti-inflammatory agents.^4^ The current standard of care for AH is treatment with prednisolone to drastically lower inflammation;^133^ however, prednisolone is not effective in most patients and increases the risk for secondary infections.^132^ Monoclonal antibody therapies targeting inflammatory cytokines, including TNFα and IL1β, have received considerable interest. Monoclonal antibodies against TNF (infliximab) are not effective therapies, at least in part due to the dual role of TNF in both generating inflammation and promoting hepatocyte health.^157^ Thus, there has been a shift to the current focus on the use of monoclonal antibodies against IL1 (canakinumab) in clinical trials (NCT03775109). Anakinra, a small molecular IL1 receptor antagonist, has been tested in one clinical trial in combination with zinc and pentoxifylline, but the results have yet to be published.^146^ Anakinra, again in combination with zinc, is currently being tested in a large multicenter clinical trial in the US (NCT04072822).

Chemokines are also key therapeutic targets for interrupting inflammation in AH patients. Preclinical studies with CVC, a dual inhibitor of CCR2 and CCR5, have shown promising results,^82^ and MIF098, an inhibitor of the pluripotent cytokine/chemokine MIF, is also a promising agent for reducing chronic ethanol-induced liver injury in mice.^52,158^

As alternatives to strategies that directly break the cycle of proinflammatory cytokine and chemokine signaling, strategies that promote anti-inflammatory responses and hepatocyte regeneration are also of interest. The most well studied are strategies involving IL22,^156,159^ which has been shown to be promising from the perspectives of safety and efficacy.^156^

### Combination therapies

Interestingly, many of the ongoing or registered clinical trials on AH involve the use of combination therapies. For example, the Defeat ASH (DASH) consortium utilized a combination of zinc to improve gut health and anakinra/pentoxifylline to inhibit inflammation.^146^ Multiple registered clinical trials have proposed examining the influence of G-CSF with N-acetylcysteine or prednisolone.^147^ Given the many tissues that are impacted by chronic alcohol consumption, combined therapeutic approaches targeting multiple pathways may indeed be the best strategies for future interventions.

### Behavioral interventions

There is a growing appreciation in the hepatology community for the important place of behavioral therapy in the treatment of patients with ALD.^160,161^ Behavioral science and psychology are now being integrated into hepatology consultations to better serve patients with this important aspect of therapy. Importantly, behavioral scientists are taking advantage of innovative approaches, such as the use of avatars and mobile apps, to better treat patients with AUD.^162^ Notably, some therapeutic agents being tested in preclinical models of ALD, such as inhibitors of PDE4,^163,164^ are also potential therapeutic targets for decreasing alcohol consumption behaviors.^165,166^

## Immunological mechanisms of NAFLD

### Triggers of inflammation in NAFLD

#### Hepatocyte death in NAFLD

Hepatocyte death is one of the key triggers of liver inflammation in NAFLD and NASH progression.^15^ Different cell death modes play different important roles in NAFLD progression. Apoptosis is considered a key participant in NASH, and research has shown that hepatocyte apoptosis leads to increased release of DNA fragments from apoptotic bodies, stimulates HSC activation and causes fibrosis.^167,168^ Emerging evidence supports the idea that hepatocyte apoptosis induced by death receptors such as TRAIL promotes the recruitment of immune cells and activates the immune system by stimulating the secretion of EVs and multiple chemokines.^169^ In addition, KCs produce TNF, TRAIL, and FAS ligands through phagocytosis of apoptotic bodies, which subsequently promotes hepatocyte apoptosis and causes hepatitis and fibrosis.^170^ Necrosis, a regulatory type of programmed cell death, is mediated by a complex of RIP1 and RIP3. The expression of RIP3 in NASH patients and mouse models is elevated and associated with JNK activity and inflammation. In addition, hepatic inflammation and liver fibrosis are significantly reduced in mice with methionine- and choline-deficient (MCD) diet-induced Rip3 deficiency.^171^ Pyroptosis, a newly described type of caspase 1-dependent cell death, can activate the inflammasome, and these processes result in continuous release of cytoplasmic contents.^172^ The release of IL-1β and IL-18 into the circulation activates the immune system.^173,174^ Many lines of evidence suggest that alteration of autophagy in hepatocytes and nonparenchymal cells (KCs and HSCs) contributes to NASH pathogenesis.^175^ For example, Kwanten et al. showed that autophagy deficiency in hepatocytes leads to apoptosis and inflammation in mice through unfolded protein response (UPR) regulation.^176^ In addition, the weakening of liver autophagy leads to insufficient removal of damaged mitochondria, and oxidative stress and release of mitochondrial factors trigger hepatocyte apoptosis and liver inflammation in NASH.^177^ Inhibiting IL-1 signaling reduces hepatocyte death and liver fibrosis, inflammation, and steatosis in mouse models of NASH.^178^

#### Gut microbiome

The gut and liver communicate via tight bidirectional links through the biliary tract, portal vein and systemic circulation.^179^ Many studies have shown that the gut microbiota plays a key role in the pathogenesis of NAFLD. Loomba et al. characterized the gut microbiota of NAFLD patients through whole-genome macrogenomics and found increased levels of *Escherichia coli* and *Bacteroides vulgatus* in patients with advanced fibrosis.^180^ In obese children with and without NASH, Zhu et al. observed a significant increase in gut microbial ethanol production as the number of alcohol-producing bacteria (especially *E. coli*) in the microbiota increased;^181^ however, the levels of endogenous ethanol are very low, and the role of endogenous ethanol in NAFLD remains controversial. Gut barrier dysfunction increases bacterial translocation and may promote NAFLD progression. Recent studies have demonstrated that damage to the gut vascular barrier driven by the microbiota leads to the transfer of bacteria or bacterial products into the blood circulation, which is a prerequisite for liver inflammation and NASH development.^182^ Patients with NAFLD also have intestinal inflammation and decreased numbers of CD4^+^ and CD8^+^ T lymphocytes in the intestinal mucosa, which are associated with increased cytokine secretion and disruption of tight junctions.^183^ Rahman et al. found that mice lacking junctional adhesion molecule (JAM)-A had increased intestinal permeability and bacterial translocation to the liver, which drives hepatitis and NASH. Furthermore, the development of hepatitis and NASH was eliminated after administration of local intestinal antibiotics, confirming an important role of the microbiota in driving liver inflammation in NASH.^184^ The gut microbiota in infants of obese mothers increases inflammation and susceptibility to NAFLD.^185^ Whether the association of gut microbiome alterations with NAFLD parameters shown in human studies is causal remains to be seen.

Bile acids (BAs) regulate the metabolism of lipids and carbohydrates via activation of farnesoid X receptor (FXR) and G protein-coupled BA receptor 1 (TGR5). Abnormal BA metabolism promotes hepatitis and fibrosis.^186,187^ BAs can regulate lipid synthesis by stimulating FXR; in addition, BAs and the gut microbiota can regulate each other and subsequently promote the development of NAFLD and NASH.^188^ Many FXR and TGR5 activators have been detected in BA analogs to reduce hepatic steatosis and inflammation, such as obeticholic acid, which has been recognized as a new treatment for NASH and cholestatic diseases.^189,190^ Suppressed hepatic bile acid signaling despite elevated production of primary and secondary BAs in NAFLD.^191^

#### Adipocyte death and inflammation

Adipose tissue is the largest endocrine organ, and it has been revealed that crosstalk between adipose tissue and liver tissue affects systemic metabolism and insulin resistance. Several studies have shown that beyond its role as a major supplier of fatty acids to the liver,^192^ adipose tissue plays a key role in regulating NASH development by secreting adiponectin, leptin, TNF, and IL-6.^193,194^ Leptin can promote inflammation by triggering KC activation and stimulating KCs to release TNFα.^195^ On the other hand, adiponectin inhibits the proliferation of HSCs.^196^ In addition, some lipid moieties (palmitic acid, ceramide) released by adipocytes also hinder the function of the endoplasmic reticulum (ER) and mitochondria, causing cell stress and even hepatocyte death.^197^ In addition to affecting hepatocytes, lipotoxic substances can activate infiltrating macrophages and KCs.^198^ Calprotectin (S100A8 and S100A9) from adipose tissue may activate KCs through TLR4 and NLRP3 signaling.^199^ Similarly, TNFα released by adipose tissue leads to hepatocyte death and activates KCs through JNK pathways.^200^ Finally, adipocyte death is associated with obesity, which plays an important role in the pathogenesis of NASH. A recent study clearly demonstrated that adipocyte death predominantly induces liver injury and inflammation in a model of acute adipocyte death via activation of CCR2+ macrophages and elevation of epinephrine and norepinephrine levels to induce lipolysis.^74^ Current understanding of the role of adipose-derived EVs in metabolic homeostasis and diseases: communication from the distance between cells/tissues.^201^ The novel adipokine gremlin 1 antagonizes insulin action and is increased in type 2 diabetes and NAFLD/NASH.^202^

#### Diet and nutrient intake

Inadequate vitamin and fiber content in the diet, as well as simple carbohydrates, saturated fat, and excessive cholesterol, are associated with NASH development. Unhealthy diets, sedentary lifestyles, and even weight gain itself are major risk factors for NAFLD, independent of baseline body mass index.^203^ High fructose intake promotes intestinal inflammation, which in turn increases endotoxin release and epithelial dysfunction and reduces the levels of tight junction proteins independent of dietary fat content and energy intake.^204^ Fructose promotes NASH through several mechanisms, including upregulation of hepatic inflammatory genes and downregulation of hepatic mitochondrial metabolite levels.^205^ Increased dietary intake of saturated fatty acids induces the UPR, resulting in ER stress and apoptosis.^206^ In addition, dietary cholesterol intake has been found to be associated with NAFLD risk and severity.^207^ In the NAFLD mouse model, high-cholesterol diet feeding promotes a strong inflammatory response in the liver. Among the mechanisms are mitochondrial dysfunction, increased ROS production, and induction of ER stress via activation of free cholesterol and hepatocyte death pathways.^208,209^ Free cholesterol also accumulates in KCs and HSCs to activate liver inflammation and fibrosis.^210^

### Inflammatory cells in NAFLD

#### KCs and infiltrating macrophages

In the context of NAFLD, KCs are a major source of cytokines and chemokines, including TNFα, IL-1β, and CCL2.^211,212^ Depletion of KCs/macrophages through the use of gadolinium chloride or phosphonic acid liposomes in animals improves liver steatosis and hepatic inflammation, suggesting the important role of KCs/macrophages in NAFLD.^213,214^ Binding of LPS to TLR4 on the KC surface in NAFLD activates the NF-κB pathway, resulting in massive release of cytokines and thus contributing to the progression of inflammation and fibrosis.^215^ In addition, hepatocyte apoptosis is significantly increased in NASH, which can activate KCs through phagocytosis of apoptotic bodies.^216^ In animal models, KCs promote the early stage of NASH by increasing TNF-α and CCL2 production.^217^ Furthermore, activation of NLRP3 in KCs promotes IL-1β secretion, thereby boosting the development of NASH.^218^ Recent studies have demonstrated that stimulator of IFN genes (STING, also referred to as TMEM173), which is a receptor that recognizes released DNA and triggers innate immune activation, functions as a mtDNA sensor in KCs and subsequently promotes NF-κB-dependent inflammation in NASH.^219,220^ Furthermore, KCs are involved in regulating lipid metabolism and insulin sensitivity in hepatocytes, increasing the accumulation of triglycerides in hepatocytes and reducing fatty acid oxidation and insulin responsiveness, while neutralizing antibodies against TNF-α can alleviate KC-induced liver injury.^214,221^ Recently, researchers have revealed that KC homeostasis is impaired during NASH, which alters the liver response to lipids as well as KC ontogeny.^84^ The landscape of intercellular crosstalk in healthy and NASH livers was revealed by single-cell secretome gene analysis.^222^ RORα induces KLF4-mediated M2 polarization in liver macrophages that protect against NASH.^223^

Similar to KCs, the recruitment of bone marrow‐derived macrophages is also a crucial event in NAFLD. Monocyte infiltration is dependent on chemokine receptors such as CCR2 and CXCR3.^213,224,225^ In MCD diet- or obesity-induced NASH, inhibition of CCL2 or CCR2 decreases macrophage recruitment, thereby ameliorating hepatic inflammation and fibrosis.^226^ Likewise, inflammation is significantly improved in the CXCR3^−/−^ mouse model.^225,227^ Lymphocyte antigen 6C2 (LY6C2)^+^ monocyte infiltration, primarily via CCR2–CCL2-mediated recruitment, is a critical pathogenic event that promotes steatohepatitis and subsequent fibrosis progression in NASH.^226,228^ Although presumably some recruited macrophages differentiate into tissue-resident macrophages, studies have suggested that infiltrating monocytes and KCs are morphologically different and transcriptionally diverse, emphasizing the presence of two major hepatic macrophage subsets in NAFLD.^229^

#### Neutrophils

The neutrophil-to-lymphocyte ratio (NLR) is significantly independently correlated with advanced inflammation and fibrosis and is suggested to be a valid diagnostic biomarker for NASH and terminal fibrosis in NAFLD patients.^230,231^ Infiltrating neutrophils in the liver secrete cytokines and active molecules to alter the progression of NASH. Interaction between neutrophils and other immune cells is also of great concern; for example, studies have found that adipose tissue macrophages worsen liver damage by enhancing neutrophil recruitment.^232^ Furthermore, the levels of myeloperoxidases (MPOs) secreted by neutrophils are increased in NASH patients, and MPOs have been shown to be toxic to macrophages, thereby contributing to the progression of inflammation and insulin resistance.^233^ Deletion of the key neutrophilic enzymes (MPO or elastase) markedly reduces liver inflammation and improves insulin sensitivity in mice.^234,235^ In neutrophil and HSC cocultures, neutrophils can trigger HSC activation via MPO, thereby promoting liver fibrosis. Interactions between neutrophils and HSCs may also play important roles in the synergistic effects of obesity and binge drinking on liver fibrosis.^236^ Adipose tissue macrophages induce hepatic neutrophil recruitment and macrophage accumulation in mice.^232^ Increased proteinase 3 and neutrophil elastase plasma concentrations are associated with NAFLD and type 2 diabetes.^237^

Recent studies have demonstrated that overexpression of CXCL1 or IL-8 can induce hepatic neutrophil infiltration and promote the progression of fatty liver to NASH in high-fat diet (HFD)-fed mice, which is mediated via the p47^Phox^-dependent production of ROS by neutrophils.^155^ Neutrophils can release neutrophil extracellular traps (NETs) to control infection, and in humans, elevated NET markers in serum are associated with NASH severity; similarly, reducing NET release improves liver inflammation and NASH-related HCC in mouse models.^234^

#### DCs

Liver DCs, as APCs, internalize antigens and transport them to regional lymph nodes to form a bridge between the innate and adaptive immune responses.^238,239^ The role of DCs in NAFLD remains unclear due to contradictory data. Henning et al. reported that DC depletion significantly enhances hepatic inflammation and fibrosis, suggesting that DCs inhibit NASH progression.^240^ Other studies have demonstrated that DCs contribute only in a minor way to CCL4-induced models of fibrosis.^241^ In contrast, other studies have used models of MCD-induced NASH to show that DCs play proinflammatory roles in disease processes, and depleting DCs reduces proinflammatory cytokine and chemokine expression, thereby ameliorating liver fibrosis.^238,242^ The findings of a study by Connolly et al. suggest that DCs promote the progression of liver fibrosis and inflammation in NASH.^243^ The conflicting nature of these results may be rooted in the use of different mouse models or the heterogeneity of liver DCs,^244,245^ but further studies are needed to clarify the role of DCs in NAFLD. Differential activation of hepatic invariant NKT cell subsets plays a key role in the progression of NASH.^246^ Murine CD103+ DCs protect against steatosis progression towards steatohepatitis.^245^ Myeloid cells in the liver and bone marrow acquire a functionally distinct inflammatory phenotype during obesity-related steatohepatitis.^247^

#### T lymphocytes

T cells are key components of the adaptive immune system and exist in multiple differentially active subsets: the T-helper (Th) cell subset, the regulatory T (Treg) cell subset, the cytotoxic T (Tc) cell subset, and several innate T-cell subsets. Th cells assist macrophages, effector T cells, and B cells to eliminate pathogens and infected cells. The levels of Th1-associated cytokines (e.g., IFNγ) in the liver are elevated in NAFLD, whereas the levels of Th2-associated cytokines, including IL-4, IL-5, and IL-13, are decreased.^248^ Little data exist regarding the roles of the Th1 and Th2 subsets in NAFLD; however, the roles of Th17 cells in NAFLD have been extensively studied over the last two decades, and accumulating data suggest that Th17 cells can release Th17 cytokines to activate KC-mediated secretion of proinflammatory cytokines, including IL-6, IL-1, and TNF, thus aggravating liver inflammation and progressive fibrosis.^248,249^ Additionally, it has been shown that IL-17 reduces hepatic, muscle and adipose tissue insulin sensitivity.^249,250^ Moreover, the heterodimeric integrin receptor α4β7 regulates CD4^+^ T-cell recruitment to inflamed tissues; blocking such α4β7-mediated recruitment of CD4^+^ T cells to the intestine and liver not only attenuates hepatic inflammation and fibrosis but also improves metabolic dysfunction associated with NASH.^251^

One study has shown that adoptive transfer of Treg cells can alleviate HFD-induced hepatic inflammation because of a decrease in hepatic TNFα expression.^252^ The opposite is observed in human liver steatosis, in which most available studies suggest that liver Treg cell numbers are increased.^248^ These findings could imply a dual role for Treg cells. Toll-like receptor-7 signaling promotes NASH by inhibiting regulatory T cells in mice.^253^ Memory CD4^+^ and CD8^+^ T-cell numbers are increased while naïve T-cell numbers are decreased in the peripheral blood of NAFLD patients.^254^ Moreover, the number of infiltrating CD8^+^ T cells in the portal vein is elevated in NAFLD patients and is associated with the severity of hepatic inflammation.^255,256^ Furthermore, activation of Tc cells promotes the secretion of proinflammatory cytokines, including IFNγ and TNFα.^257^ Nishimura et al. have shown that Tc cells are essential for macrophage recruitment and adipose tissue inflammation because they secrete chemotactic molecules, thus demonstrating the key role of Tc cells in NASH development.^258^ Other studies have shown that Tc cell-derived perforin participates in the mechanism regulating liver inflammation and thus plays a protective role in the development of NASH.^259^ In addition, a recent study revealed that Tc cells interact synergistically with NKT cells to promote the progression of NASH and increase the incidence of NASH-related HCC.^257^

#### NKT cells

NKT cells can rapidly respond to antigen recognition by secreting cytokines, including IFNγ, IL-4, and IL-13. NKT cells can accumulate in fatty tissue,^260^ but reversible decreases in NKT cell numbers aid in recovery from hepatic inflammation.^254,260^ NKT cell prevalence within the liver varies during the course of disease depending on the signals present. Studies have confirmed that IL-12 secreted by KCs can lead to NKT cell depletion.^261^ Similarly, NAFLD-associated hepatic NKT cell depletion induces apoptosis by activating Tim-3 expressed on terminally differentiated T cells.^262^ Moreover, studies have found that NKT cells can attenuate hepatocyte steatosis and liver inflammation, thereby relieving NAFLD progression.^263,264^ Other studies, however, contradict the above conclusions, suggesting that NKT cells do not affect or play a role in promoting NASH.^265–267^ A small increase in NKT cell numbers has been found in the adipose tissues of HFD-fed mice.^268^ In addition, some experiments have shown that NKT cells can alleviate liver inflammation and insulin resistance in mice; however, there is also some evidence that NKT cells can aggravate obesity and hepatic inflammation.^260,269,270^ Thus, NKT cells may both stimulate and suppress inflammatory responses, which needs further exploration.

### Emerging mechanisms in NAFLD

#### EVs

A number of studies have demonstrated that EVs contribute to key processes involved in the pathogenesis and progression of NAFLD, including angiogenesis, fibrosis, and inflammation.^271–273^ The EVs secreted by hepatocytes can promote the expression of proinflammatory cytokines and polarize hepatic macrophages to the M1 phenotype.^274–276^ Mixed-lineage kinase 3 (MLK3) induces lipid-treated hepatocytes to release EVs containing CXCL10 to recruit macrophages. The total numbers of plasma EVs and the numbers of EVs containing CXCL10 in MLK3-knockout mice fed a NASH-inducing diet are lower than those in wild-type mice.^277^ Moreover, EVs released from hepatocytes can contribute to hepatic recruitment of monocyte-derived macrophages by promoting monocyte adhesion via integrin β1 (ITGβ1)-dependent mechanisms.^278^ Hepatocytes release ceramide-enriched inflammatory EVs by activating IRE1A, and EVs recruit monocyte-derived macrophages to the liver, resulting in inflammation in mice with steatohepatitis.^279^ Thus, lipotoxic injury of hepatocytes boosts the release of EVs and activates macrophages to promote hepatic inflammation, which plays an important role in triggering NAFLD. These findings provide strong support for the development of EVs as biomarkers, and EVs are also potential therapeutic targets and tools.^271^

#### Inflammasome

The inflammasome machinery has a two-step activation requirement that results in cytosolic assembly of its components and cleavage of downstream substrates with production of active IL-1β, IL-18, and active GSDMD. Activation of the inflammasome machinery is necessary for a wide range of sterile inflammatory processes, and the details of the biochemistry of inflammasome activation have been widely reviewed.^280,281^ Here, we will focus on the evidence for a role of inflammasome activation in ALD and NAFLD.

The total loss of several individual inflammasome components (purinergic receptors 2 × 7,^282^ NLRP3,^283^ and caspase-1^284,285^) has been shown to reduce steatosis, inflammation and fibrosis in a number of models of diet-induced NASH and ALD.^79,286,287^ Furthermore, liver histology in NASH is improved by the NLRP3 inhibitor MCC950.^288^ Among all types of liver cells, liver macrophages, as expected, have the highest expression of inflammasome components, with the greatest evidence of inflammasome activation and requirement for NASH and ASH.^79^ Collectively, these findings form a substantial dataset supporting the requirement of a functional NLRP3 inflammasome pathway for the development of full NASH and ASH liver pathology, with KCs being the main responsible cell type. It has been demonstrated that whole-body forced expression of constitutively active NLRP3 inflammasomes in the context of liver pathology induces neutrophilia and inflammation in many tissues, including the skin and large joints. The same experiment also demonstrated hepatocyte death and HSC activation. Hepatocyte death is a particular type termed pyroptosis, which has many of the features of apoptosis, including DNA damage and terminal deoxynucleotidyl transferase-mediated dUTP nick‐end labeling (TUNEL) positivity. However, in stark contrast to apoptosis, in which the intracellular contents are retained during cell death, pyroptosis features membrane pore development and subsequent release of intracellular contents, which stimulates a local inflammatory response.^289^ The roles of the NLRP3 inflammasome machinery in other liver cell populations are not fully resolved. Hepatocytes have very low levels of expression of inflammasome components, and there have been reports of active caspase 1 and IL-1β production by hepatocytes, but this is not a widely reported phenomenon.^290^ Primary murine HSCs as well as LX-2 cells, an immortalized human stellate cell line, express all components of the NLRP3 inflammasome, and its activation using monosodium urate crystals, a potent signal two inflammasome activator, results in a phenotypic switch from quiescent to myofibroblast collagen-producing cells.^291^ Constitutive activation of the NLRP3 inflammasome in HSCs also results in a marked increase in the number of cells positive for α smooth muscle actin, a key marker of activated HSCs, and spontaneous development of liver fibrosis.^292^ Thus, these data strongly support the concept that the NLRP3 inflammasome plays a direct role in liver fibrotic responses with significant implications related to the development of novel strategies for the treatment of liver fibrosis.

#### MtDNA

The concept of danger signals was developed before the identification of such signals but has subsequently been found to be valid. Currently, over twenty molecules fulfilling this function have been identified and are grouped under the general term DAMPs.^293,294^ The defining feature of these molecules is functional, as they are all released by cells in response to stress or injury, and they subsequently elicit responses from other cells that aim to protect the cells from pathogens and reestablish homeostasis (through defense, repair, or regeneration). One notable aspect of DAMPs is their structural diversity: they range from nucleic acids to proteins to small molecules (such as ATP) and even crystals (uric acid).^295^ DAMP-induced activation of the innate immune response is known to be protective against pathogens, but one trade-off is that in the context of sterile injury, DAMP-induced inflammation paradoxically increases tissue injury. Such sterile inflammation-driven tissue injury is seen in many organs but is notably severe in the liver, which has a very active innate immune response and an inactive adaptive response.^4^ This phenomenon of sterile inflammation-induced injury has many important clinical consequences for conditions including ischemia/reperfusion injury, acetaminophen toxicity, ALD and NAFLD.^16,296,297^

Among the many DAMPs, nucleic acids, particularly DNA, are strong mediators of sterile inflammation. DNA has the desirable features of being intracellular, resistant to breakdown by damaging signals such as ROS and able to activate intracellular pathways such as the cGAS-STING pathway and extracellular pathways such as the TLR9 pathway. The ability of mtDNA to be released by hepatocytes during acetaminophen toxicity and by muscle cells after crush injury has been found to activate an immune response via TLR9.^298,299^ Increases in serum DNA and particularly in mtDNA have been observed in NASH- and acetaminophen-induced models of liver injury and in patients.^300,301^ Experimental models have yielded evidence that the DNA receptor TLR9 plays an important role, as revealed in TLR9-deficient mice.^298,302,303^ MtDNA has some features that make it a more effective DAMP than nuclear DNA, including hypomethylation compared to nuclear DNA, possibly at CpG motifs that are known to be potent patterns for activation of absent in melanoma 2 (AIM2), cGAS, and TLR9.^304,305^ A further feature that enhances the functional DAMP ability of mtDNA is the high levels of ROS generated in mitochondria, which result in oxidation of deoxyribonucleosides; an oxidized derivative of deoxyguanosine, 8-Oxo-dG, is the major product of DNA oxidation.^306^ These qualitative changes mean that quantification of DNA in the serum does not entirely reveal its efficacy as a DAMP. This is further complicated by the association of DNA with other molecules, such as mitochondrial transcription factor A (TFAM) and high-mobility group protein 1 (HMGB1), which greatly increases its ability to activate TLR9.^307,308^ The main cell type activated via TLR9 ligands is hepatic macrophages. Ligand signaling results in the production of a range of cytokines, including IL-1 and IL-18, via activation of the inflammasome. In addition to macrophages, neutrophils have also been demonstrated to be activated by TLR9 ligands, and this activation results in both neutrophil activation and upregulation of mir-223 via the IKKα and NF pathways. Subsequently, mir-223-mediated downregulation of signaling occurs via inhibition of IKK, resulting in a negative feedback loop to limit the degree of inflammation.^309^

In addition to inflammation, TLR9 activation on HSCs results in HSC activation; a lack of TLR9 greatly reduces HSC activation and liver fibrosis.^168^ Recently, it has been demonstrated that serum mtDNA levels are elevated in patients with NASH and correlated with the degree of liver fibrosis. Furthermore, mtDNA can activate HSCs in vitro and enhance liver fibrosis when injected in vivo.^310^ A possible reason for the different degrees of fibrotic responses in mice, and speculatively in humans, may be the efficiency of removal of apoptotic hepatocytes by liver macrophages. Slow and ineffective removal results in greater release of DAMPs such as mtDNA. As suggested by the interaction of mtDNA with TFAM and HMGB1, multiple interactions can occur, and DAMPs do not circulate in isolation. Many DAMPs, including mtDNA, are present inside EVs that originate from hepatocytes and contain a complex of proteins and miRNAs. These proteins can modulate SMA activation by downregulating mRNA in a miRNA-dependent manner and activating KCs in a TLR9-dependent manner.^9^

In summary, cell stress and death by metabolic excess and alcohol result in the release of a number of DAMPs, many inside EVs, that can stimulate the activation of KCs and HSCs to initiate and maintain inflammation and fibrosis. Downstream of DAMP receptors, a number of pathways, including the inflammasome pathway, are activated, which results in cleavage of caspase and production of the proinflammatory molecules IL-1b and IL-18 and can also induce cellular pyroptosis.

### Inflammatory targets for NASH

#### Inflammatory Targets for the Treatment of NAFLD

Currently, there are no approved therapies for NAFLD and NASH. The pathogenesis of NAFLD is thought to originate from hepatic caloric overload, which in turn leads to hepatocyte metabolic and oxidative stress and initiates the inflammatory responses detailed above. Thus, potential therapeutic interventions for NAFLD could target each of these steps.

#### Metabolic target modulators

Initial therapeutic approaches for NAFLD aimed at targeting the metabolic process by utilizing systemic insulin sensitizers. Pioglitazone, a PPARγ agonist, has been demonstrated in several studies to treat NASH. In the phase IIb PIVENS trial,^311^ nondiabetic patients received pioglitazone for 96 weeks. Histological improvement was seen in 34% of pioglitazone-treated subjects compared to placebo-treated subjects. Similar findings were seen after up to 36 months of treatment in a randomized trial enrolling diabetic and prediabetic patients.^312^ Thiazolidinediones (TZDs) have multiple modes of action and can exert them in multiple tissues. Adipose tissue is likely a main target tissue, as evidenced by the association between improved adipose tissue insulin sensitivity with pioglitazone treatment and the hepatic histological response.^313,314^ Importantly, despite its primary metabolic target, pioglitazone treatment improves histological inflammation scores (defined by the presence of inflammatory cell infiltration) and fibrosis, confirming that metabolic dysfunction is the main driver of disease progression.

Although TZDs are often thought of as PPARγ agonizts, they can also activate the hepatic mitochondrial pyruvate carrier complex.^315^ In an attempt to decrease PPARγ-mediated side effects, MSC-0602, a PPARγ-sparing TZD, has been investigated. Despite promising results in an animal model,^316^ a recent phase IIb clinical trial failed to demonstrate efficacy in human NASH.^317^ Similarly, elafibranor, a medication targeting PPARα and PPARδ, has shown some benefit in a phase IIb study;^318^ however, a phase 3 trial in NASH has not shown benefit in an interim analysis, leading to study discontinuation.

Another successful approach has been treatment with glucagon-like peptide 1 (GLP-1) receptor agonizts (GLP-1RAs). Endogenous GLP-1 is an incretin hormone that modulates pancreatic insulin secretion, insulin secretion, and peripheral insulin sensitivity and delays gastric emptying.^319^ In the small LEAN phase IIa trial, NASH resolution was achieved by 39% of nondiabetic NASH patients treated for a year with liraglutide, a once-daily GLP-1RA, compared to 9% of patients treated with placebo (*p* = 0.02).^320^ Recently, semaglutide, another GLP-1RA, was reported to lead to NASH resolution in 59% of subjects, although the results have not yet been published in a peer-reviewed manuscript. GLP-1RAs modulate multiple metabolic pathways and induce weight loss. Importantly, there is no evidence for a GLP-1 receptor on human hepatocytes;^321^ thus, any benefit seen in the liver is due to extrahepatic effects.

Recently, there has been an intense focus on the use of FXR agonizts to treat NAFLD. As detailed above, FXR is the intracellular sensor for BAs in the liver and intestines, and its activation decreases hepatic gluconeogenesis, de novo lipogenesis and steatosis.^322,323^ Obeticholic acid (OCA), a modified BA, is a potent FXR agonist that has been studied in patients with NASH. In the phase II FLINT trial, histological improvement was seen in 45% of patients treated with OCA for 72 weeks compared to 21% of controls, and NASH resolved in 22% of patients.^324^ In the 18-month interim analysis of the phase III REGENERATE trial, NASH resolution with treatment was not superior to that with placebo, but a modest benefit was seen in the fibrosis regression endpoint (23 vs. 12% in placebo).^325^ This trial is ongoing. Several other FXR agonizts are also in advanced clinical trials, and the results are awaited.

Other agents targeting global metabolic pathways have shown benefit in phase II clinical trials, including analogs of fibroblast growth factor 19 (FGF-19),^326^ fibroblast growth factor 21^327^ and thyroid hormone receptor β agonizts.^328^ In addition to agents targeting global pathways, there are also agents specific to hepatic lipid metabolism. Firsocostat is a liver-targeted acetyl-CoA carboxylase (ACC) inhibitor that effectively decreases hepatic de novo lipogenesis and steatosis.^329,330^ However, firsocostat was not effective in decreasing liver fibrosis in the phase II ATLAS trial, either as monotherapy or in combination with other agents.

#### Targeting inflammatory pathways

Although most studies developing medications have focused on initiating metabolic processes, several attempts have been made to target downstream inflammatory and injury pathways. Vitamin E, a lipid-soluble antioxidant, was tested in the PIVENS trial,^311^ which demonstrated a histological response in 43% of subjects and resolution of NASH in 36% of subjects after 96 weeks of treatment. Importantly, vitamin E is the only medication to date that has been associated with improved clinical outcomes, leading to increased transplant-free survival and decreased hepatic decompensation.^331^ Given the known role of oxidative stress in NASH pathogenesis, it is not surprising that vitamin E treatment leads to improvements in histological ballooning and inflammation scores. Surprisingly, it also leads to decreased steatosis,^311^ which is thought to be upstream of its site of action. This effect has recently been shown to be directly related to its antioxidant effect and to be mediated by a decrease in hepatic de novo lipogenesis through inhibition of late SREBP-1c maturation.^332,333^ Currently, vitamin E appears to be a safe, effective and inexpensive therapy that is readily available.

Beyond the relatively nonspecific vitamin E, several other therapeutic targets that appear to be more specific to NASH-associated inflammation have been explored. As detailed above, apoptosis appears to play a major role in the pathogenesis of NASH. A key regulator of the apoptosis pathway is apoptosis signal-regulating kinase 1 (ASK1), a kinase that is activated in hepatocytes by oxidative stress, ER stress, or TNFα and leads to apoptosis and fibrosis deposition. In addition, ASK1 is essential for the development of TNFα-mediated insulin resistance and steatosis in mouse models.^334^ These findings have led to the development of selonsertib, an ASK1 inhibitor. Selonsertib was recently studied in two phase III trials in patients with NASH and advanced fibrosis (STELLAR 3) or cirrhosis (STELLAR 4) but failed to meet its primary endpoint of improvement of fibrosis.^335^ Another antiapoptotic agent is emricasan, an oral pancaspase inhibitor that has high first-pass metabolism,^336^ rendering it relatively liver targeted. The ENCORE-NF trial evaluated emricasan in patients with noncirrhotic NASH, aiming to improve fibrosis, while the ENCORE-PH study examined emricasan in patients with NASH cirrhosis and severe portal hypertension, aiming to improve the hepatic venous pressure gradient (HVPG). In both trials, emricasan treatment was not superior to placebo treatment,^337,338^ and further drug development was halted.

As previously discussed, the release of chemokines such as CCL2 and CCL5 is crucial for the recruitment of lymphocytes to the liver in NASH and in the progression of fibrosis. CVC, a CCR2/CCR5 antagonist, was studied in the CENTAUR phase IIb trial in patients with NASH and fibrosis. After 1 year of treatment, the primary endpoint of improvement in NASH histology was not met (16% of patients vs. 19% in placebo).^338^ However, fibrosis was improved by 20% (compared to 10% in the placebo group, *p* = 0.02). Based on these findings, the ongoing phase III AURORA trial aims to determine the efficacy of CVC in decreasing fibrosis and liver-related clinical events.^339^

#### Genetic targets

Several genetic loci have been implicated in the pathogenesis of NAFLD and NASH and have the potential to be targeted therapeutically. The I148M mutation in patatin-like phospholipase domain-containing protein 3 (PNPLA3) is strongly associated with hepatic steatosis, inflammation, and fibrosis.^340,341^ In mice fed a NASH-inducing diet, treatment with antisense oligonucleotide (ASO) directed against PNPLA3 leads to improvements in steatosis. Furthermore, in mice carrying the human I148M mutant, ASO treatment improves inflammation and fibrosis.^342^ Based on these findings, AZD2693, a PNPLA3-targeting ASO, is currently being evaluated in a phase I trial in healthy overweight subjects and in patients with NASH who are PNPLA3-I148M homozygous.

Recently, several variants in the 17β-hydroxysteroid dehydrogenase 13 (HSD17B13) gene were found to be associated with NASH inflammation, injury and fibrosis^343,344^ and with decreased development of HCC in individuals with ALD.^345,346^ HSD17B13 is a lipid droplet-associated protein with retinol dehydrogenase enzymatic activity.^344^ Importantly, the variants that lead to loss of enzymatic function are genetically associated with improved outcomes. Although the protective phenotype could not be replicated in a mouse knockout model,^347^ several pharmaceutical companies have announced the development of an HSD17B13-targeted ASO, and at least one phase I clinical trial has been initiated.

In summary, there is a great need for effective medications to treat NAFLD and NASH. To date, the most benefit has been seen with agents that target the global dysregulated metabolic profile, including medications such as GLP-1RAs that have no direct liver effects. In contrast, medications aimed at inflammatory targets downstream of the metabolic load have not shown strong benefits, with the exception of vitamin E and possibly CVC. Combination therapies may be needed to unleash the full potential of these agents.

## Conclusions and future perspectives

ALD and NAFLD are the two major types of chronic liver diseases worldwide, and multiple drivers are involved in their pathogeneses (Figs. 1, 2). Among them, inflammation is believed to play a key role in promoting the progression from simple fatty liver to more severe forms of liver injury, such as steatohepatitis, cirrhosis, and HCC. Although many inflammatory mediators have been identified, as discussed above, the key factor(s) that drive the progression of ALD and NAFLD have not been clarified in patients and may differ from patient to patient. Future studies to identify these key inflammatory drivers will not only enhance our understanding of fatty liver disease pathogeneses but also help us discover novel and effective therapeutic interventions for the treatment of ALD and NAFLD. Several inflammatory therapeutic targets have been or are currently being evaluated in clinical trials for the treatment of AH and NASH. We expect more clinical trials using inflammatory mediators as therapeutic targets for the treatment of fatty liver diseases to be conducted in the future.
